# Gut microbiota alterations and microbial translocation in HIV/SARS-CoV-2 co-infected patients

**DOI:** 10.3389/fcimb.2026.1688580

**Published:** 2026-02-12

**Authors:** Xuan Yan, Xinyu Zhang, Lin Wang, Wei Song, Tangkai Qi, Zhenyan Wang, Yang Tang, Jianjun Sun, Shuibao Xu, Junyang Yang, Yueming Shao, Youming Chen, Jiangrong Wang, Jun Chen, Renfang Zhang, Li Liu, Yinzhong Shen

**Affiliations:** Department of Infectious Diseases and Immunology, Shanghai Public Health Clinical Center, Fudan University, Shanghai, China

**Keywords:** co-infection, gut microbiome, HIV, metagenomics, microbial translocation, SARS-CoV-2

## Abstract

**Objective:**

To characterize gut microbiome alterations and microbial translocation in human immunodeficiency virus (HIV)/severe acute respiratory syndrome coronavirus 2 (SARS-CoV-2) co-infected patients and identify microbial signatures associated with COVID-19 severity.

**Methods:**

In this cohort study, blood and fecal samples from 38 HIV/AIDS patients (20 SARS-CoV-2 co-infected [PC group]; 18 SARS-CoV-2-negative [NC group]) were analyzed. The PC group was stratified by COVID-19 severity: mild-to-moderate (PC1, n=13), severe-to-critical (PC2, n=3), and mixed infections (PC3, n=4). Serum lipopolysaccharide (LPS), soluble CD14 (sCD14), and zonulin levels were measured to assess microbial translocation and gut barrier integrity. Fecal metagenomic profiling was performed via whole-genome shotgun sequencing (Illumina NovaSeq/HiSeq).

**Results:**

Co-infected patients exhibited significantly elevated plasma LPS (78.09 *vs* 48.72 pg/mL, p=0.032) and sCD14 (2667 *vs* 1927 ng/mL, p=0.0015) compared to controls. Although no differences in α-diversity or overall taxonomic abundance were observed between the PC and NC groups, 329 PC-unique and 216 NC-unique microbial species were identified. Nine genera demonstrated diagnostic potential for co-infection [Area Under the Curve (AUC), >0.7] with *Akkermansia* showing the highest predictive value (AUC = 0.811). Critically, *Blautia* abundance was significantly reduced in severe-to-critical cases (PC2) versus mild-moderate cases (PC1, p=0.043) and controls (NC, p=0.006). Besides, our function prediction for gut microbiota suggested that SARS-CoV-2 may exacerbate lipid metabolic dysregulation in HIV-infected individuals.

**Conclusions:**

HIV/SARS-CoV-2 co-infection is characterized by heightened microbial translocation and species-specific microbiota alterations rather than global dysbiosis. *Blautia* depletion may correlate with COVID-19 severity.

## Introduction

Advanced age, immunosuppression, male sex, and comorbidities have been recognized as risk factors for severe outcomes after severe acute respiratory syndrome coronavirus 2 (SARS-CoV-2) diagnosis, and concerns about human immunodeficiency virus (HIV)/SARS-CoV-2 co-infection remain substantial (Triant and Gandhi, 2021; Braunstein et al., 2023). A large population-based cohort study in South Africa demonstrated a twofold higher risk of COVID-19 mortality in people living with HIV (PLWH) than in uninfected individuals (Risk factors for coronavirus disease 2019 (COVID-19) death in a population cohort study from the Western Cape Province, South Africa, 2021). Consistent with this finding, a study in England also reported a significantly elevated risk of COVID-19 mortality among PLWH after adjusting for demographic and lifestyle factors (Bhaskaran et al., 2020). Furthermore, analysis of outcomes among hospitalized COVID-19 patients in the UK indicated that HIV-positive status was associated with an increased risk of 28-day mortality (Geretti et al., 2021).

Despite apparent differences in the diseases they cause, the two viruses share significant parallels in their pathogenic mechanisms (Illanes-Álvarez et al., 2021). In HIV infection, chronic cytokine release drives persistent inflammation, which is associated with increased intestinal permeability and bacterial translocation—pathological features that persist despite antiretroviral therapy (ART) (Vujkovic-Cvijin et al., 2020; Illanes-Álvarez et al., 2021). In contrast, COVID-19 is characterized by an acute cytokine surge that contributes directly to clinical manifestations. This response attracts inflammatory cells from the bloodstream, amplifying tissue damage (Liu et al., 2020). Consequently, systemic inflammation markers serve as key prognostic indicators in COVID-19 (Liu et al., 2020; Soy et al., 2020). While both infections are known to disrupt immune and inflammatory responses, whether co-infection causes unique changes compared to PLWH without COVID-19 requires further investigation.

Additionally, a key commonality between HIV and SARS-CoV-2 infections is manifested through alterations in the gut microbiota (Illanes-Álvarez et al., 2021). Evidence indicates that SARS-CoV-2 patients developing cardiac complications exhibit elevated intestinal permeability and inflammasome activation, suggesting a heart-gut axis in COVID-19 pathogenesis (Moccia et al., 2020). Similarly, HIV infection disrupts host-microbiota-immune interactions, characterized by microbiota alterations—specifically an increase in pro-inflammatory bacteria and decrease in homeostatic species—which collectively promote bacterial translocation and dysregulated immune responses (Lozupone et al., 2013; Marchetti et al., 2013a; Dillon et al., 2014). Nevertheless, the exact mechanisms by which intestinal microbiota influence disease progression during HIV and SARS-CoV-2 coinfection have not yet been fully elucidated.

In this study, we investigated COVID-19-associated alterations in gut microbiota composition among PLWH. Our findings demonstrate that SARS-CoV-2-infected PLWH exhibited significantly increased gut microbial translocation and distinct microbiome compositional changes compared to SARS-CoV-2-negative PLWH. Notably, some of these microbial alterations showed a graded association with COVID-19 disease severity. This research provides important mechanistic insights into gut microbiome dysbiosis in HIV/SARS-CoV-2 coinfection, advancing our understanding of disease pathogenesis and progression. The identified microbial signatures may facilitate development of novel diagnostic biomarkers and therapeutic strategies for managing this vulnerable patient population.

## Materials and methods

### Participants

This investigation encompassed patients with HIV and SARS-CoV-2 coinfection admitted to the Department of Infection and Immunity at the Shanghai Public Health Clinical Center (SPHCC), Fudan University from February 2023 to March 2024, as the experimental group (PC group). HIV/AIDS patients without COVID-19 were collected during the same time period as the control group (NC group).

The inclusion criteria for the experimental group were as follows: (1) aged over 18 years old, (2) diagnosed with HIV cases in accordance with the diagnostic principles of the Chinese AIDS Diagnosis and Treatment Guidelines (Chinese guidelines for the diagnosis and treatment of human immunodeficiency virus infection/acquired immunodeficiency syndrome (2024 edition), 2024), (3) confirmed cases of COVID-19 based on the diagnostic criteria set forth in the Diagnosis and Treatment Plan for COVID-19 (Trial Version 10) (China GOoNHCotPsRo et al., 2023), which includes a positive nucleic acid test for SARS-CoV-2, (4) absence of any medical conditions that may lead to constipation or diarrhea, and (5) had not taken any probiotics, proton pump inhibitors (PPIs), or medications known to cause constipation for three months prior. The inclusion criteria for the control group were as follows: (1) aged over 18 years old, (2) diagnosed with HIV infection/AIDS, (3) negative for SARS-CoV-2 nucleic acid test, (4) absence of any medical conditions that may lead to constipation or diarrhea, and (5) had not taken any probiotics, PPIs, or medications known to cause constipation for three months prior. The exclusion criteria for both the experimental and control groups included the following: (1) pregnant women, (2) individuals with significant deviations from standard dietary practices, such as those following vegetarian or low-carbohydrate diets, (3) those suffering from severe malnutrition, active infections, or substance addiction, (4) presence of organic intestinal pathologies (e.g., intestinal tuberculosis, Crohn’s disease, or ulcerative colitis) as confirmed by either colonoscopic examination or barium enema radiography, (5) any history of surgical procedures involving the intestinal tract, and (6) individuals with comorbidities involving severe diseases of the heart, brain, liver, kidneys, hematopoietic system, or other significant primary illnesses.

### Sample collection

All participants’ peripheral blood samples in the PC group were collected on the first day of admission with a confirmed diagnosis of coinfection of HIV and SARS-CoV-2, and all participants’ peripheral blood samples in the NC group were collected on the first day of admission with a confirmed diagnosis of HIV infection. The samples were centrifuged at 2000 rpm for 10 min at 4°C, and the supernatant was frozen in a −80°C refrigerator. Fecal samples from PC and NC groups were collected at the time of hospital admission prior to medication administration and processed in the laboratory within 4 hours after collection. All fecal samples were dispensed in 2 ml Eppendorf tubes within 30 min, each tube containing 200 ± 20 mg, and immediately stored at -80°C until analysis (Zhu et al., 2022). Meanwhile, we also performed blood tests such as immune function and HIV viral load on the first day of the participants’ admission and collected the clinical indicators. All samples were collected before the participants used antibiotics.

### Enzyme-linked immunosorbent assay

The concentrations of [lipopolysaccharide (LPS)] in serum were quantified using a sandwich ELISA kit ([Catalog Number. CSB-E09945h; CUSABIO, USA]) following the manufacturer’s protocol. The concentrations of [soluble CD14 (sCD14)] in serum were quantified using a sandwich ELISA kit ([Catalog Number DC140; R&D Systems, USA]) following the manufacturer’s protocol. The concentrations of zonulin in serum were quantified using a sandwich ELISA kit ([Catalog Number D711327; BBI, China]) following the manufacturer’s protocol.

### Metagenomic sequencing and microbiota analysis

This project utilized Illumina NovaSeq/HiSeq high-throughput sequencing platforms to perform whole-genome shotgun (WGS) metagenomic sequencing. The extracted total metagenomic DNA from the microbial community was randomly fragmented into short segments. Sequencing libraries with appropriately sized inserts were constructed, and these libraries were subjected to paired-end sequencing.

Community diversity metrics (α-diversity) and between-sample dissimilarities (β-diversity) were computed using QIIME2. Differential abundance analysis was subsequently performed using linear discriminant analysis (LDA) effect size (LEfSe) method to identify taxa demonstrating both statistical significance (LDA score > 2.0, p < 0.05) and biological relevance across sample groups. Functional pathways based on the Kyoto Encyclopedia of Genes and Genomes (KEGG, http://www.genome.jp/kegg/) were used to predict the functional composition of the gut microbiota for each sample using the “search” module of the Many-against-Many sequence searching (MMseqs2) software with the sensitivity parameter set to 5.7. Images were drawn by the R (V3.5.2) package. All LEfSe analyses used the NC group as the reference group.

### Statistical analysis

Statistical analyses were performed using Stata 16.0, GraphPad Prism 8.3.1 and R 4.2.1 software. Enumeration data were expressed as mean ± standard deviation, and qualitative data were expressed as rate. Non-normally distributed variables were expressed as interquartile ranges (IQRs). The Chi-squared (χ2) test and the t-test were employed to identify statistically significant differences. Beta diversity analysis was performed using Bray–Curtis dissimilarity, and statistical significance of group differences was assessed using permutational multivariate analysis of variance (PERMANOVA) with 999 permutations. Receiver operating characteristic (ROC) curve analysis was conducted to evaluate the diagnostic performance of microbial genera in distinguishing co-infected patients, and the area under the curve (AUC) was reported with 95% confidence intervals. P<0.05 were considered statistically significant.

### Ethical approval

This study was conducted in accordance with the Declaration of Helsinki and approved by the Institutional Review Board of Shanghai Public Health Clinical Center (Ethics Approval No. 2023-S082-01). Written informed consent was obtained from all participants prior to the collection of peripheral blood and fecal samples.

## Results

### Study cohorts

Due to changes in the COVID-19 pandemic situation and patient admission strategies, a total of 38 patients were enrolled in this study during the study period. There were 20 cases in the PC group, including 19 males and 1 female, aged 49.6 ± 19.9 years. There were 18 HIV/AIDS patients without SARS-CoV-2 infection (NC group), including 17 males and 1 female, aged 43.1 ± 18.7 years. According to the classification criteria outlined in the Diagnosis and Treatment Protocol for COVID-19 (Trial Version 10) (China GOoNHCotPsRo et al., 2023), in the PC group there were 13 cases of mild-to-moderate COVID-19 (PC1 group), 3 cases of severe-to-critical COVID-19 (PC2 group), 4 cases of mixed infections (PC3 group). The PC3 group being defined as a “mixed infection” group indicates that these patients had co-infections with other respiratory pathogens alongside SARS-CoV-2. We attempted to match the main risk factors associated with HIV co-infection with SARS-CoV-2, including age, HIV RNA, CD4+T cell counts, time of HIV diagnosis and the status of ART. Following the application of χ2 tests and t-tests, these factors were found to be statistically insignificant between the HIV/AIDS patients co-infected with SARS-CoV-2 and those without SARS-CoV-2 infection (p > 0.05). All subjects were Han nationality and had similar dietary structure. **Table 1** presents the brief clinical background of the subjects involved in this study.

**Table 1:** Clinical characteristics of patients enrolled in this study.

Parameters	HIV/AIDS patients co-infected with SARS-CoV-2 (PC group)				HIV/AIDS patients without SARS-CoV-2 infection (NC group)(n=18)	P value
	Totally(n=20)	PC1 (n=13)	PC2 (n=3)	PC3 (n=4)		
Male Gender (%)	95.00	100.00	66.67	100.00	94.44	0.939
Age (years)	49.6 ± 19.9	55.0 ± 20.2	51.3 ± 18.8	30.5 ± 4.0	43.1 ± 18.7	0.306
CD4+T cell counts (cells/μL)	244.30 ± 666.20	328.00 ± 819.45	202.72 ± 329.06	224.21 ± 22.51	124.90 ± 163.30	0.458
CD4+T/CD8+T cell ratio	0.30 ± 0.52	0.41 ± 0.63	0.20 ± 0.27	0.05 ± 0.05	0.25 ± 0.43	0.758
HIV RNA (10^6 copies/mL)	0.58 ± 0.75	0.56 ± 0.72	0.27 ± 0.28	0.68 ± 0.94	0.47 ± 0.46	0.607
Proportion with >1 Year Since HIV Diagnosis (%)	15.00	15.38	33.33	0	16.67	0.888
On ART (%)	15.00	15.38	33.33	0	16.67	0.888

The Chi-squared (χ2) test and the t-test were used to compare between the PC group and NC group. p value < 0.05 is considered statistically significant. Values are mean ± SD. PC1: cases of mild-to-moderate COVID-19; PC2: severe-to-critical COVID-19; PC3: cases of mixed infections; ART: Antiretroviral Therapy.

### Differences in gut mucosal breakdown and microbial translocation between groups

**Figure 1:**
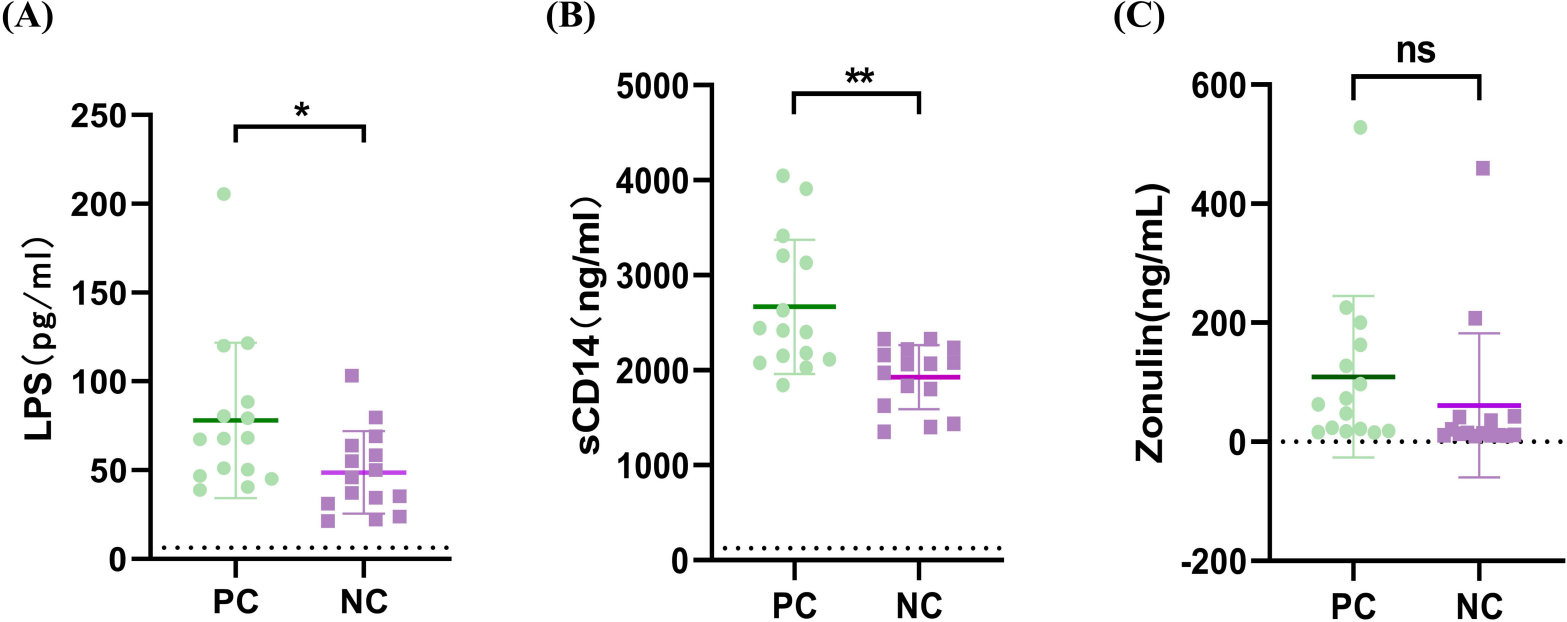
**(A-C)** Plasma levels of gut barrier dysfunction and microbial translocation markers in HIV/AIDS patients with and without SARS-CoV-2 co-infection. Plasma concentrations of lipopolysaccharide (LPS), soluble cluster of differentiation 14 (CD14), tight junction protein (zonulin). Statistical analysis: Mann-Whitney U test. PC, HIV/AIDS patients co-infected with SARS-CoV-2; NC, HIV/AIDS patients without SARS-CoV-2 infection. *p < 0.05; **p <0.01.

To indirectly assess participants’ intestinal microbial translocation, we quantified the levels of microbial translocation markers LPS (Ouyang et al., 2023), monocyte activation biomarkers sCD14 (Martínez et al., 2021), and intestinal barrier integrity indicators zonulin (Dirajlal-Fargo et al., 2020) in plasma. These markers were measured using commercially available ELISA kits according to the manufacturer’s protocols. Due to greater challenges in collecting clinical blood samples compared to fecal specimens from patients, we obtained only 15 reliable plasma samples each from the PC group (including 10 cases from the PC1 group, 2 cases from the PC2 group, and 3 cases from the PC3 group) and the NC group for ELISA analysis. The baseline characteristics of these 30 clinical samples are presented in **Table 2**. We observed significantly higher levels of LPS in HIV/AIDS patients co-infected with SARS-CoV-2 than individuals without SARS-CoV-2 [78.09 pg/ml (IQR: 46.81–88.41) versus 48.72 pg/ml (IQR: 31.24–63.91); p =0.0320; **Figure 1A**]. Besides, plasma sCD14 levels were markedly increased in HIV/AIDS patients co-infected with SARS-CoV-2 (median 2667 ng/mL, IQR 2113-3208) when compared to individuals without SARS-CoV-2 (median 1927 ng/mL, IQR 1626-1927; p=0.0015; **Figure 1B**).We did not detect significant differences in zonulin levels between the two groups but a trend to higher levels in HIV/AIDS patients co-infected with SARS-CoV-2 than individuals without SARS-CoV-2 [109.30 ng/ml (IQR: 18.43–163.20) versus 61.08 ng/ml (IQR: 10.84–41.40); p =0.3131; **Figure 1C**]. However, no significant differences were found in the three indicators among the PC1, PC2, and PC3 groups (p > 0.05).

**Table 2:** Clinical characteristics of patients enrolled in ELISA.

Parameters	HIV/AIDS patients co-infected with SARS-CoV-2 (n=15)	HIV/AIDS patients without SARS-CoV-2 infection(n=15)	P value
Male Gender (%)	93.33	86.67	0.543
Age (years)	53.3 ± 20.2	46.5 ± 20.4	0.287
CD4+T cell counts (cells/μL)	158.20 ± 163.39	216.90 ± 177.50	0.353
CD4+T/CD8+T cell ratio	0.47 ± 0.55	0.80 ± 1.4	0.417
HIV RNA (10^6 copies/mL)	0.35 ± 0.89	0.30 ± 0.42	0.772
Proportion with >1 Year Since HIV Diagnosis (%)	6.67	6.67	0.684
On ART (%)	6.67	6.67	0.684

The Chi-squared (χ2) test and the t-test were used to compare the two groups. p value < 0.05 is considered statistically significant. Values are mean ± SD.

### Richness, diversity, and composition of gut microbiota among groups

**Figure 2:**
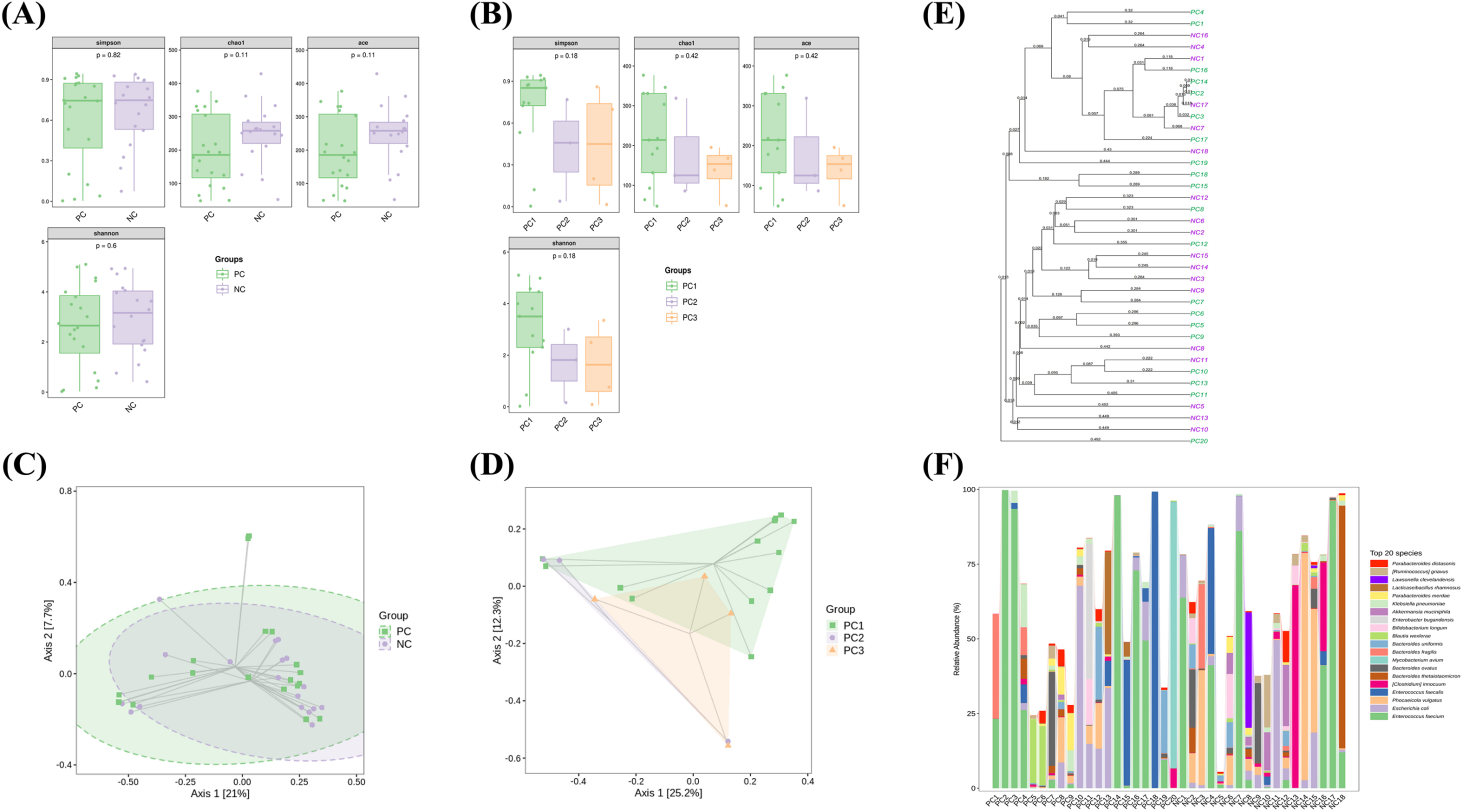
Gut microbiota diversity and composition in HIV/AIDS patients with and without SARS-CoV-2 co-infection. **(A, B)** Alpha diversity metrics (Chao1, ACE, Shannon, Simpson) showing no significant differences between the PC (HIV/AIDS patients co-infected with SARS-CoV-2) and NC (HIV/AIDS patients without SARS-CoV-2 infection) groups **(A)**, or among PC subgroups stratified by COVID-19 severity (PC1, mild-to-moderate; PC2, severe-to-critical; PC3, mixed infections) **(B)**. **(C, D)** Principal coordinates analysis (PCoA) based on Bray-Curtis dissimilarity, illustrating significant β-diversity patterns in microbial community composition between PC and NC groups **(C)** and among PC subgroups **(D)**. **(E)** Unweighted pair group method with arithmetic mean (UPGMA) clustering analysis of gut microbiota profiles across samples. **(F)** Stacked bar plots depicting the relative abundance of the top 20 microbial species in each group. Statistical significance was assessed using Kruskal-Wallis test.

Rarefaction curves based on the observed species (Sobs) index were generated to assess sequencing depth. All groups reached near-asymptotic saturation, confirming adequate sequencing coverage. Neither the comparison between PC and NC groups nor the comparisons among PC subgroups (PC1, PC2, PC3) revealed statistically significant differences in α-diversity, as assessed by richness estimators (Chao1, ACE) and diversity indices (Shannon, Simpson) (**Figures 2A, B**). The PERMANOVA test revealed significant differences in β-diversity between the PC and NC groups (p<0.05). Principal coordinates analysis (PCoA) based on Bray-Curtis dissimilarity (**Figures 2C, D**) and unweighted pair group method with arithmetic mean (UPGMA) analysis (**Figure 2E**) revealed significant β-diversity patterns in microbial community composition between sample groups. **Figure 2F** displays stacked bar plots showing the composition of dominant microbial taxa (top 20 most abundant species) across all samples.

### Gut microbiota differences between groups

**Figure 3:**
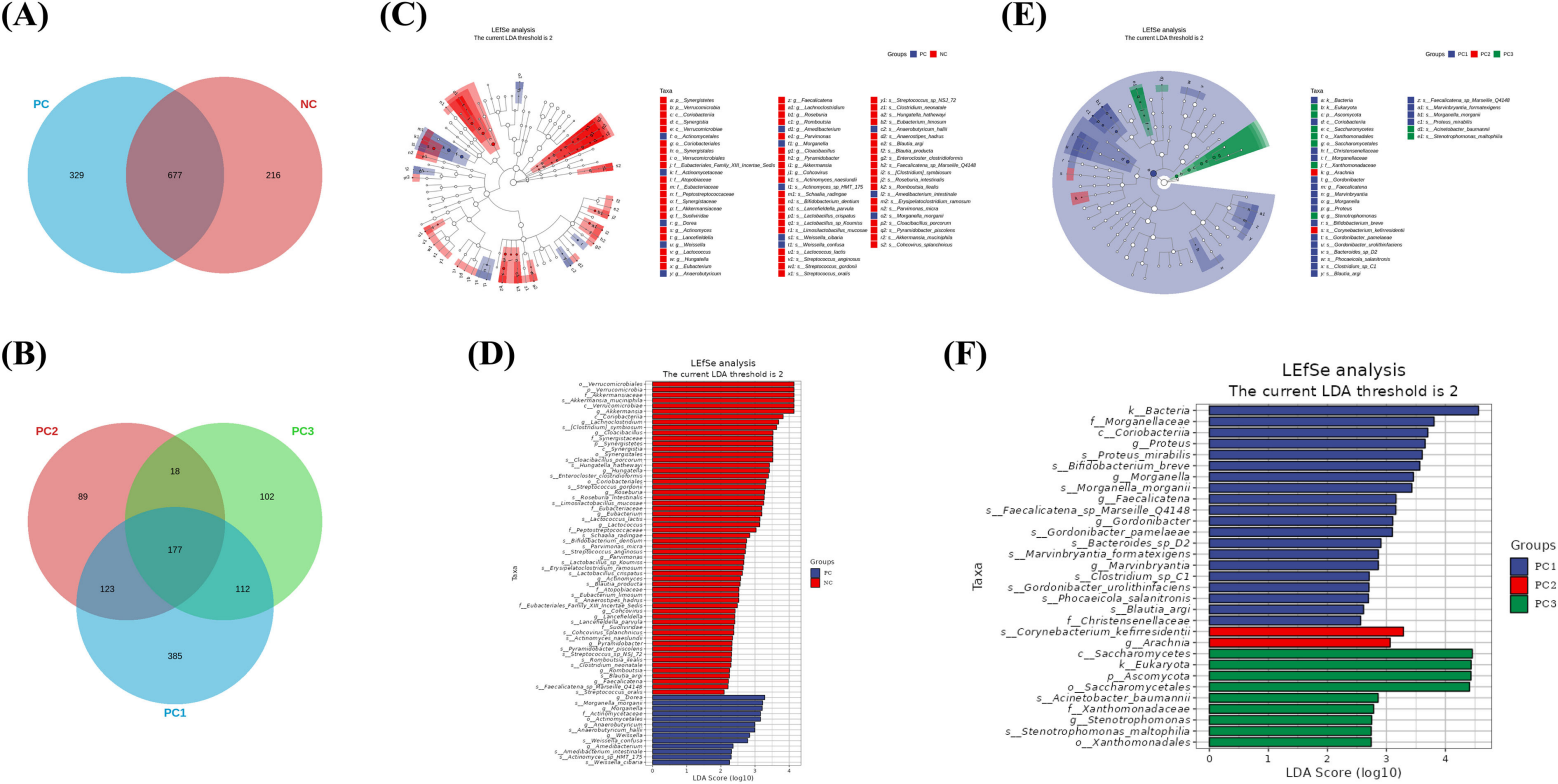
Gut microbial signatures and diagnostic potential in HIV/SARS-CoV-2 co-infection. **(A, B)** Venn diagrams showing shared and unique microbial species between **(A)** the PC (HIV/AIDS patients co-infected with SARS-CoV-2) and NC (HIV/AIDS patients without SARS-CoV-2 infection) groups and **(B)** across PC subgroups (PC1, mild-to-moderate; PC2, severe-to-critical; PC3, mixed infections). **(C)** LEfSe cladogram analysis identifying discriminant intestinal flora (LDA score > 2, p < 0.05) between PC and NC groups. Circles from the inside out indicate phylogenetic levels from phylum to genus. **(D)** LEfSe analysis of intestinal flora. LEfSe identified the taxa with the greatest differences in abundance between PC and NC groups. **(E)** LEfSe cladogram analysis identifying discriminant intestinal flora (LDA score > 2, p < 0.05) of PC subgroups (PC1, PC2, PC3). Circles from the inside out indicate phylogenetic levels from phylum to genus. **(F)** LEfSe analysis of intestinal flora. LEfSe identified the taxa with the greatest differences in abundance of PC subgroups (PC1, PC2, PC3). Statistical significance was determined by Mann-Whitney U or Kruskal-Wallis tests. LEfSe analysis was performed with the NC group as the reference.

A Venn diagram was constructed to visualize microbial species shared among groups and those uniquely present in each group. A core microbiota of 677 species was not associated with SARS-CoV-2 infection status in HIV-infected individuals (**Figure 3A**). Additionally, a subset of 177 species persisted across all COVID-19 severity strata (mild-to-severe) and mixed infections phenotypes (**Figure 3B**).

Metagenomic sequencing revealed no statistically significant differences in gut microbial composition between PC and NC groups at any taxonomic level (phylum to species), nor among PC subgroups (all p >0.05). To identify specific communities associated with HIV and SARS-CoV-2, we compared the gut microbiota composition between groups using LEfSe analysis. For PC and NC group, LEfSe analysis revealed 29 discriminant features (LDA >2, p <0.05, **Figures 3C, D**) at phylum (n = 2), family (n = 8), and genus (n = 19) levels. For PC1, PC2 and PC3 group, LEfSe analysis revealed 11 discriminant features (LDA >2, p <0.05, **Figures 3E, F**) at phylum (n = 1), family (n = 3), and genus (n = 7) levels. Notably, *Blautia_argi* exhibited significantly lower relative abundance in severe-to-critical COVID-19 cases (PC2) versus both mild-to-moderate cases (PC1) (p = 0.043, LDA = 2.612) and COVID-19-negative controls (NC) (p=0.006, LDA = 2.252), implying a possible link to clinical deterioration. Additionally, no other taxa were observed to show significant alterations in severe-to-critical COVID-19 cases.

### Identification of HIV/SARS-CoV-2 co-infection based on intestinal flora

**Figure 4:**
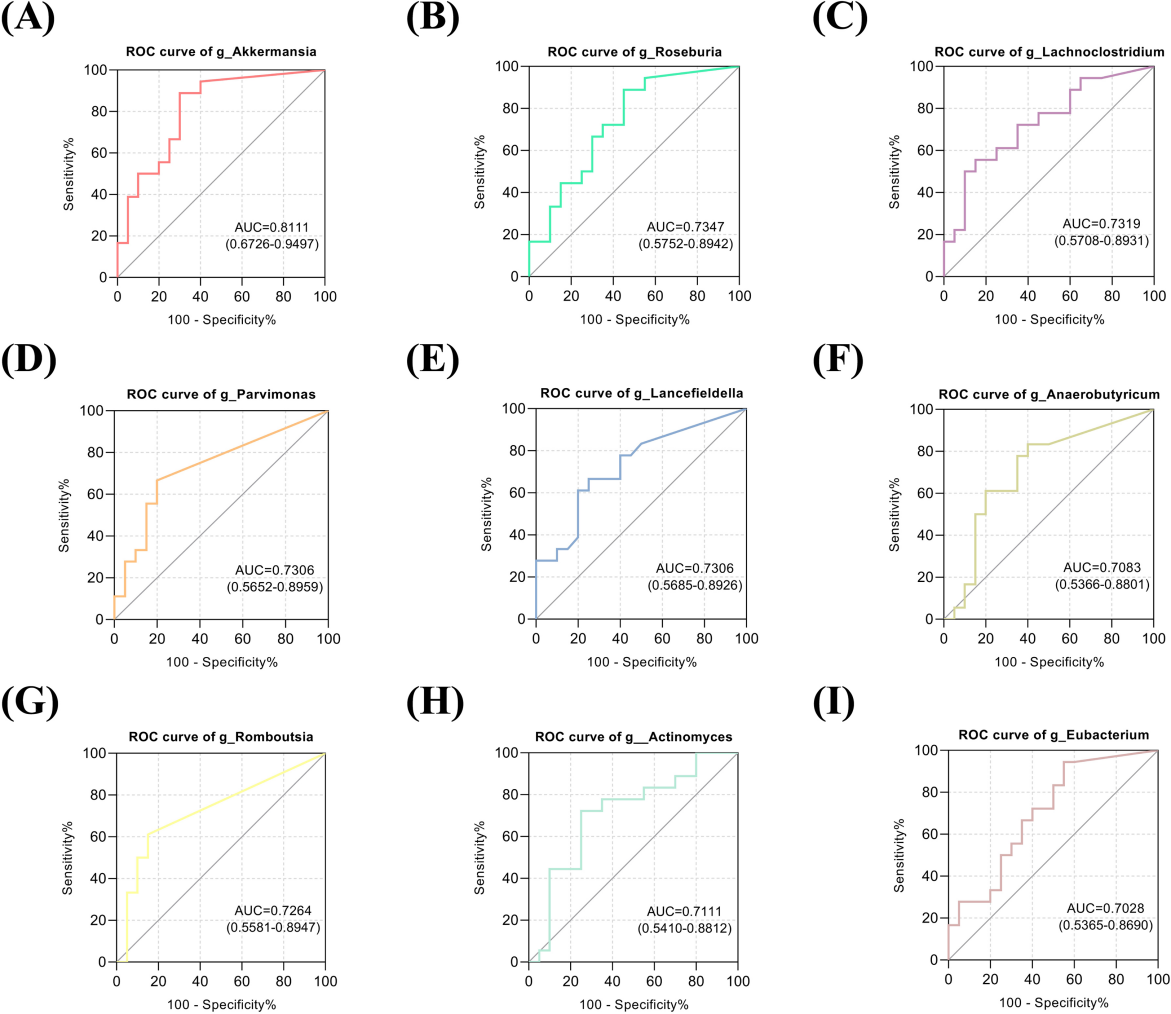
Receiver operating characteristic (ROC) curve analysis of gut microbial genera for distinguishing HIV/AIDS patients co-infected with SARS-CoV-2 from HIV/AIDS patients without SARS-CoV-2 infection. **(A-I)** ROC curves demonstrating the diagnostic performance of nine bacterial genera with area under the curve (AUC) values > 0.7. *Akkermansia* (AUC = 0.811) exhibits the highest predictive accuracy, followed by *Roseburia* (AUC = 0.735), *Lachnoclostridium* (AUC = 0.732), *Parvimonas* (AUC = 0.731), *Lancefieldella* (AUC = 0.731), *Romboutsia* (AUC = 0.726), *Actinomyces* (AUC = 0.711), *Anaerobutyricum* (AUC = 0.708), and *Eubacterium* (AUC = 0.703).

To evaluate the discriminatory potential of gut microbiota in identifying HIV/SARS-CoV-2 coinfection, we employed a systematic modeling approach. Bacterial taxa were initially selected based on LDA effect size, followed by performance evaluation using AUC metrics. The optimal classification model was subsequently identified through this validation process. Notably, our analysis revealed several bacterial genera with significant discriminatory power (AUC >0.7) for distinguishing between SARS-CoV-2-positive and SARS-CoV-2-negative individuals: *Akkermansia* (AUC = 0.811), *Roseburia* (0.735), *Lachnoclostridium* (0.732), *Parvimonas* (0.731), *Lancefieldella* (0.731), *Anaerobutyricum* (0.708), *Romboutsia* (0.726), *Actinomyces* (0.711), and *Eubacterium* (0.703) (**Figure 4**). Based on the aforementioned LEfSe analysis, we also found that the genus *Akkermansia* was significantly reduced in the PC group. At the family level, we observed a significant decrease in the abundance of *Akkermansiaceae* in the PC group. This family primarily consists of the genus *Akkermansia*, and its representative species, *Akkermansia muciniphila*, is a known beneficial bacterium. Its reduction may be associated with impaired intestinal barrier function. Our results similarly showed a significant decrease in the species *Akkermansia muciniphila* within the PC group (**Figure 3D**). These findings demonstrate that a gut microbiota-based classification model effectively differentiates HIV patients with concurrent SARS-CoV-2 infection from those without SARS-CoV-2 co-infection.

### Function prediction for gut microbiota in HIV and SARS-CoV-2 co-infected patients

**Figure 5:**
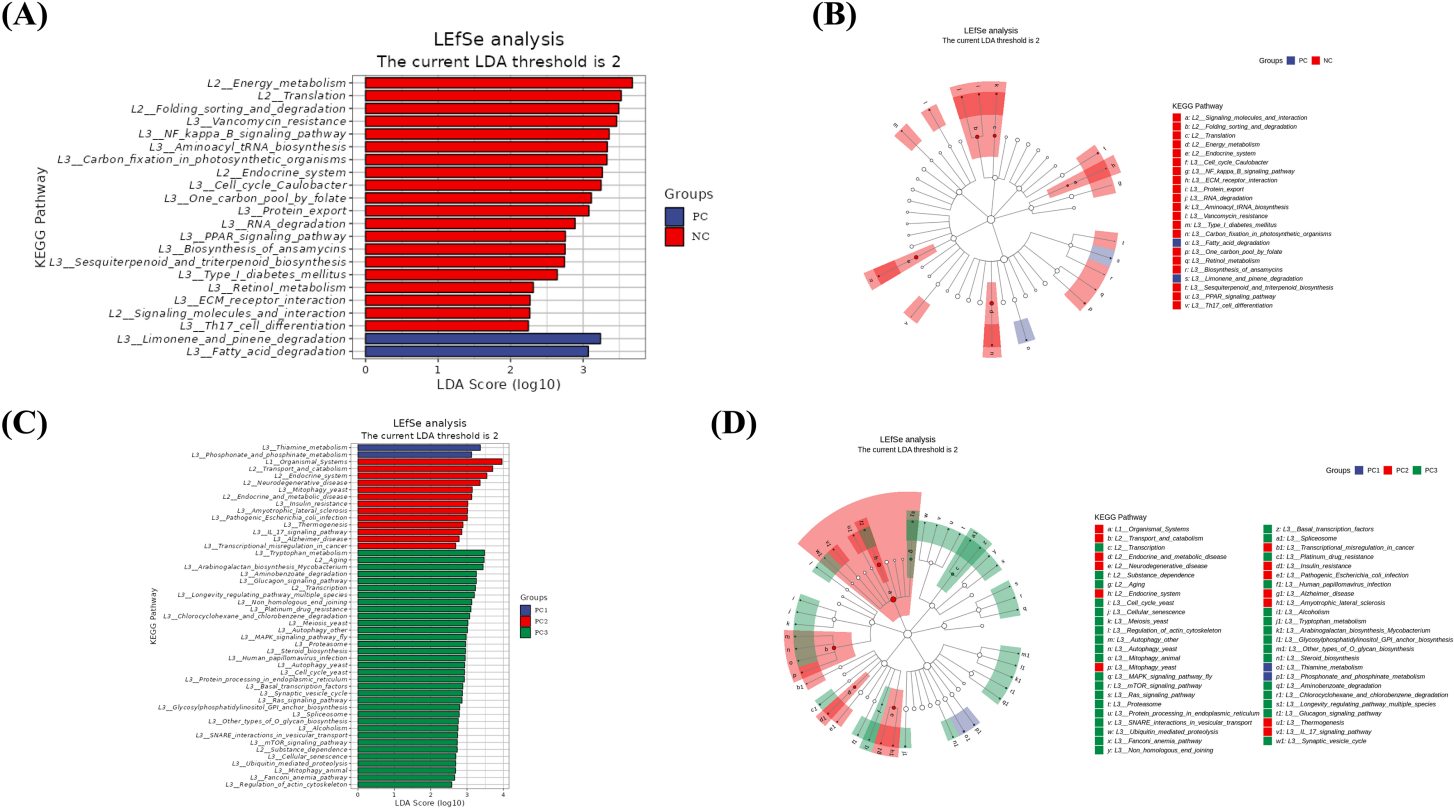
Functional pathway analysis of gut microbiota in HIV/SARS-CoV-2 co-infected patients. **(A, B)** LEfSe analysis highlighting functional pathways enriched in the PC group (HIV/AIDS patients co-infected with SARS-CoV-2) compared to the NC group (HIV/AIDS patients without SARS-CoV-2 infection). **(C, D)** Enrichment of pathways in severe-to-critical COVID-19 cases (PC2 subgroup), suggesting systemic metabolic dysregulation linked to disease severity. LDA score threshold > 2.0, p < 0.05. LEfSe analysis was performed with the NC group as the reference.

LEfSe analysis can not only identify differential species but also delve into functional groups at different levels to uncover those with intergroup differences. By performing LEfSe analysis on the functional abundance table of KEGG database entries across samples, it was found that functional categories related to Limonene_and_pinene_degradation and Fatty_acid_degradation were more abundant in the PC group samples (**Figures 5A, B**). The upregulation of Fatty_acid_degradation and Limonene_and_pinene_degradation in PC group samples highlights SARS-CoV-2-induced perturbations in lipid homeostasis, which may further complicate metabolic outcomes in HIV-positive individuals. In severe-to-critical COVID-19 cases among PLWH (PC2 group), the enrichment of Organismal_Systems and Transport_and_catabolism pathways underscores a synergistic viral impact on systemic metabolism, warranting further investigation into lipid dysregulation (**Figures 5C, D**).

## Discussion

With the advent of advanced microbiome technologies, alterations in the gut microbiota during infectious diseases have become a major research focus. Our study demonstrates that HIV/SARS-CoV-2 co-infected patients exhibit two hallmark pathophysiological features: heightened microbial translocation and species-specific gut microbiota alterations without evidence of global dysbiosis. Of note, *Blautia* depletion correlated with COVID-19 severity.

Our findings demonstrate that compared to PLWH without SARS-CoV-2 infection, those coinfected with SARS-CoV-2 exhibit elevated gut translocation markers, as evidenced by significantly increased levels of LPS and sCD14 and an upward trend in zonulin. Microbial translocation - the passage of bacterial products across the compromised intestinal epithelial barrier - represents a significant contributor to persistent immune activation in HIV infection (Marchetti et al., 2013b). Specifically, in HIV-infected individuals, elevated levels of LPS and its induced monocyte activation marker sCD14 show significant correlations with multiple immune activation indicators (Ouyang et al., 2023). Interestingly, SARS-CoV-2 infection also induces increased intestinal permeability and compromises epithelial barrier integrity, particularly for patients admitted to the Intensive Care Unit (ICU) (Oliva et al., 2021; Zhang et al., 2025). Additional studies indicate that recovered COVID-19 patients exhibit significant decreases in plasma interleukin-6 (IL-6) and sCD14 levels, demonstrating resolution of systemic inflammation and response to bacterial translocation (Brooks et al., 2024). Furthermore, IL-6 shows significant positive correlations with sCD14 (Brooks et al., 2024). Moreover, HIV infection triggers a redistribution of zonulin from the gut into the bloodstream, where its elevated levels are linked to intestinal CD4+ T-cell depletion and inflammation (Augustin et al., 2024). Notably, despite effective viral suppression through antiretroviral therapy, HIV-infected individuals exhibit persistent systemic immune activation, with microbial translocation products now recognized as key contributors to this phenomenon (Sandler and Douek, 2012; Dirajlal-Fargo et al., 2020). Given these findings, our findings suggests that SARS-CoV-2 infection may lead to increased intestinal permeability, disruption of the gut barrier, enhanced microbial translocation and dysbiosis of gut microbiota. However, this is only indirect evidence of gut microbiota translocation. Without analyzing the plasma microbiome, we have not provided direct evidence that SARS-CoV-2 infection exacerbates gut microbial translocation in HIV-infected individuals.

In our preliminary study, we observed no significant differences in gut microbial diversity among PLWH with SARS-CoV-2 co-infection, as assessed by α-diversity metrics. Similarly, a separate investigation of HIV/Pneumocystis coinfection found no changes in species diversity (Zhu et al., 2022). Plus, our findings display a significant difference in gut microbial β-diversity between the PC and NC groups. This indicates that SARS-CoV-2 co-infection altered the gut microbiota in PLWH by changing its types and compositional structure, without altering its overall quantity or internal richness. This restructuring might occur through a dynamic shift involving the suppression of taxa dominant in the NC group and the proliferation of previously rare or exogenous species. Meanwhile, gut microbiome dysbiosis in COVID-19 manifests as diversity loss and *Akkermansia* overgrowth (Bernard-Raichon et al., 2022). Together, these findings suggest that while overall microbial composition remains stable during coinfection, the relative abundance of specific taxa may undergo significant shifts as the disease progresses. Beyond gut-specific effects, the gut-lung axis refers to the bidirectional pathophysiological interactions between the gastrointestinal and respiratory systems during disease progression, which may contribute to the pathogenesis and clinical outcomes of various conditions, including microbial translocation (Dumas et al., 2018). The lower gastrointestinal tract has been proposed as a potential source of pulmonary bacterial populations in critically ill patients. For instance, in sepsis and acute respiratory distress syndrome, bacterial translocation from the gut to the lungs may occur due to barrier dysfunction (Dickson et al., 2016; Liu et al., 2022). Based on these mechanistic insights, we hypothesized that PLWH who develop respiratory symptoms of varying severity following SARS-CoV-2 infection may exhibit gut microbiota alterations. Therefore, in our metagenomic sequencing analysis, we specifically included several severe COVID-19 cases with hypoxemia, after excluding co-infections with other pulmonary pathogens. Consistent with our hypothesis, the results confirmed characteristic shifts in bacterial taxon abundance in the fecal samples of some severe COVID-19 cases.

Our study identified nine bacterial genera with significant discriminatory power (AUC >0.7) for distinguishing between SARS-CoV-2-negative and SARS-CoV-2-positive PLWH, among which *Akkermansia* demonstrated the highest discriminatory capacity (AUC = 0.811). Emerging evidence reveals that distinct changes in the taxonomic and functional profiles of gut microbiota and associated metabolites are frequently observed across different stages of diseases, such as intracranial aneurysms (Sun et al., 2024). Building on this observation, gut microbes and their metabolic derivatives may serve as biomarkers for disease onset and progression. Our study identifies SARS-CoV-2 coinfection as a factor associated with gut microbiota alterations, including depletion of *Akkermansia*, among PLWH. These alterations may be relevant to COVID-19 clinical outcomes in this susceptible population. Historically, since its discovery and identification 20 years ago, extensive research has demonstrated that deficiency or reduced abundance of *Akkermansia_muciniphila* - this symbiotic bacterium - is associated with various diseases, including inflammatory conditions and responses to cancer immunotherapy (Cani et al., 2022). As an abundant resident member of the gut microbiota in humans and animals, the probiotic effects of *Akkermansia* - including metabolic modulation, immune regulation, and gut barrier protection - have been extensively investigated (Zhai et al., 2019). Dysbiosis in *Akkermansia_muciniphila* abundance has been associated with various pathologies, notably metabolic syndrome and autoimmune disorders (Zhai et al., 2019). Given these cumulative findings, *Akkermansia*, as a beneficial gut microbe, still requires further investigation to elucidate its precise role and mechanisms in HIV/SARS-CoV-2 co-infection.

In our study, we found a progressive decline in the abundance of *Blautia_argi* across clinical severity groups: highest in SARS-CoV-2-negative, intermediate in mild-to-moderate COVID-19 cases, and lowest in severe-to-critical COVID-19 patients. Notably, *Blautia*, an anaerobic bacterial genus with probiotic properties, is ubiquitously present in mammalian intestines and feces (Liu et al., 2021). This commensal microbe exhibits robust probiotic effects, including bioactive compound biotransformation, host health regulation, and metabolic syndrome amelioration through short-chain fatty acid production and immune modulation (Liu et al., 2021). Importantly, beneficial gut bacteria are indispensable for colonic mucus formation and its full protective function against intestinal pathogens (Holmberg et al., 2024). Beyond gut-localized effects, existing research demonstrates that *Blautia* and its metabolites synergize with immune checkpoint inhibitors to enhance CD8+ T cell-mediated tumor cytotoxicity (Wang et al., 2024). Collectively, these studies demonstrate that *Blautia*, as a beneficial gut commensal, plays critical roles in maintaining intestinal homeostasis and systemic immune function. Our findings further suggest a potential association between *Blautia* abundance and pulmonary disease severity in PLWH following SARS-CoV-2 infection. To validate this hypothesis, future investigations should quantify *Blautia* dynamics in the lower respiratory tract to establish causal relationships with disease progression in HIV/SARS-CoV-2 coinfection.

Our LEfSe analysis of metagenomic data revealed significant enrichment of microbial taxa associated with fatty acid degradation biological processes in fecal samples from HIV/SARS-CoV-2 coinfected patients. Importantly, lipids play pivotal roles throughout the viral life cycle, with viruses actively hijacking host lipid signaling and synthesis pathways to remodel cellular lipidomes (Casari et al., 2021). Moreover, circulating lipids critically contribute to viral pathogenesis by both facilitating viral replication and triggering inflammatory responses (Schoeler and Caesar, 2019). And the gut microbiota modulates host lipid metabolism via multiple pathways: (1) production of microbial metabolites (short-chain fatty acids, secondary bile acids, and trimethylamine) and (2) release of pro-inflammatory mediators (e.g., lipopolysaccharide) that influence systemic metabolic homeostasis (Schoeler and Caesar, 2019). Specifically, during the progression of COVID-19 patients from mild to severe symptoms, insufficient expression of certain apolipoproteins has been reported (Shen et al., 2020). Most of these apolipoproteins are associated with macrophage function, which is closely linked to lipid metabolism (Shen et al., 2020). Lipid components directly regulate macrophage function by promoting their immune activity, while core macrophage processes are accompanied by changes in the lipid composition and fluidity of biomembranes (Shen et al., 2020). Supporting this notion, research indicates that lipid metabolism is a central factor in SARS-CoV-2 infection (Shen et al., 2020). And our finding implies that characteristic gut microbiota alterations during coinfection may modulate the intestinal microenvironment through metabolic pathways like lipid metabolism, potentially extending systemic metabolic impacts on the host. Consequently, lipids and lipid metabolism can serve as biomarkers for SARS-CoV-2 infection and potential therapeutic targets for COVID-19 (Song et al., 2020; Wu et al., 2020). To advance these findings, our future studies will analyze and discuss alterations in plasma lipids among HIV/SARS-CoV-2 co-infected patients by integrating metabolomics and lipidomics approaches, aiming to identify lipid metabolic pathways associated with gut microbiota. This will help uncover additional mechanisms and potential therapeutic targets underlying the onset and progression of such co-infections. It should be noted that the LEfSe and ROC analyses used in this study are exploratory, and p-values were not adjusted for multiple testing. Therefore, the identified differential microbes should be considered potential biomarkers, whose confirmation awaits future validation in larger cohorts with independent samples.

This study has several important limitations that warrant consideration. Firstly, the limited cohort size, particularly the underpowered severe-to-critical subgroup (PC2 group, n=3), significantly constrains the statistical robustness and external validity of our conclusions, thereby restricting the generalizability of the findings. Consequently, the results should be regarded as preliminary or exploratory. Due to the presence of other respiratory pathogens, the mixed infection group (PC3) cannot exclude the potential influence of these pathogens, as opposed to SARS-CoV-2, on the gut microbiota of PLWH. A notable methodological constraint is the absence of an HIV-negative control cohort, which precludes definitive attribution of observed gut microbiota alterations specifically to HIV infection versus other confounding factors. Future studies incorporating such a control group would be valuable to more precisely delineate the distinct pathogenic mechanisms of each virus. While our metagenomic analyses identified putative functional pathway modifications (e.g., fatty acid degradation), these computational predictions remain unvalidated by orthogonal experimental approaches such as targeted metabolomics or isotopic tracing studies. Furthermore, the identified microbial associations, including the severity-linked *Blautia_argi* depletion, constitute correlative observations rather than mechanistic evidence, as they lack supporting *in vitro* functional assays or *in vivo* animal model validation - particularly concerning their potential role in gut-lung axis regulation during co-infection. These limitations collectively highlight the critical need for more comprehensive studies employing adequately powered longitudinal cohorts to establish temporal relationships, integrated multi-omics approaches to connect microbial signatures with functional outcomes, and experimental validation models to confirm causal mechanisms.

## Conclusion

In summary, our study provides valuable insights into the gut microbiome alterations in HIV/AIDS patients co-infected with SARS-CoV-2. We found that these patients exhibit increased intestinal permeability and microbial translocation, as evidenced by elevated plasma levels of LPS and sCD14. Although overall microbiome diversity remains stable, specific microbial signatures such as *Akkermansia* and *Blautia_argi* are associated with co-infection and COVID-19 severity. Future research should focus on larger cohorts and explore the underlying mechanisms to better understand the impact of gut microbiota on HIV/SARS-CoV-2 co-infection. These findings suggest that the gut microbiota may play a role in disease progression and could serve as potential biomarkers for identifying co-infected patients, paving the way for better co-infection management strategies.

## Data Availability

The datasets presented in this study can be found in online repositories. The names of the repository/repositories and accession number(s) can be found in the article/supplementary material.
